# Spontaneous strategy use in children with autism spectrum disorder: the roles of metamemory and language skills

**DOI:** 10.3389/fpsyg.2015.00182

**Published:** 2015-03-04

**Authors:** James M. Bebko, Thomas Rhee, Carly A. McMorris, Busisiwe L. Ncube

**Affiliations:** ^1^Children’s Learning Projects, Department of Psychology, York UniversityToronto, ON, Canada; ^2^Holland Bloorview Kids Rehabilitation HospitalToronto, ON, Canada

**Keywords:** memory, rehearsal, language, strategy, autism, metamemory, ASD

## Abstract

Metamemory, or beliefs about one’s own memory capabilities, knowing what you know, and knowing what you don’t know, has frequently been linked to the spontaneous use of rehearsal strategies in typically developing children. However, limited research has investigated mnemonic strategy use, metamemory, or the relationship between these two cognitive processes in children with autism spectrum disorders (ASDs). The current study examined the relative strength of metamemory knowledge and language skills as predictors of rehearsal use and memory performance in individuals with ASD. Twenty-one children with ASD and 21 children in a combined comparison group were matched on chronological and verbal mental age. Over two sessions, participants completed a serial recall task, a language measure, and a metamemory questionnaire. Children were classified as rehearsers/non-rehearsers based on behavioral observations and/or verbal reports of strategy use. As expected from previous research, the comparison group had a significantly higher proportion of rehearsers than the ASD group. However, spontaneous rehearsers performed significantly better on the serial recall task than non-rehearsers, regardless of group membership. Children in the comparison group had a higher mean total score on the metamemory questionnaire than the ASD group. However, when examined by rehearsal use, participants classified as rehearsers, regardless of diagnostic group, scored significantly higher on the metamemory questionnaire than non-rehearsers. Finally, across groups, hierarchical regression analyses identified both metamemory and language proficiency as significant predictors of rehearsal strategy use. The fact that the predictors showed the same relationship across the comparison group and the ASD group implies that metamemory and language proficiency, while separate entities, are both fundamental underlying skills contributing to the emergence of rehearsal strategies, and that the results are likely generalizable to other populations with developmental challenges.

## INTRODUCTION

Autism spectrum disorder (ASD) is a neurodevelopmental disorder characterized by impairments in social and communicative domains (American Psychiatric Association [APA], 2013). ASD is also commonly associated with delays in language acquisition, sensory and perceptual difficulties, and impairments in various cognitive domains, including attention, executive control, and memory (Hill, 2004; Lord and Luyster, 2009; Solomon et al., 2009). Research on memory function in ASD is varied, with some studies reporting intact or superior performance in some aspects of memory, such as semantic memory, working memory, iconic memory, and basic serial recall, while other studies provide conflicting findings or evidence of impaired functioning in aspects such as episodic memory and free recall (Russell et al., 1996; Bebko and Ricciuti, 2000; Smith et al., 2007; Gaigg et al., 2008; Cheung et al., 2010; Southwick et al., 2011; McMorris et al., 2013; Souchey et al., 2013). Despite this research on the memory systems of individuals with ASD, there has been little research on their use of memory strategies, which has historically been characterized as inflexible or non-existent (e.g., Hermelin and O’Connor, 1975).

Rehearsal has received a considerable amount of attention in the general memory strategy research literature, perhaps due to its central role in multi-store models of memory and learning, which identify rehearsal as a key means by which information is transferred from short- to long-term memory stores (Dark and Loftus, 1976). By around 7- or 8- years of age, typically developing (TD) children generally begin to spontaneously and effectively engage in verbal rehearsal when sequentially ordered information is to be recalled, and those children who engage in rehearsal tend to display superior recall abilities compared to those who do not (e.g., Flavell, 1970; Bebko, 1984; Bebko and McKinnon, 1990; Bjorklund et al., 1992; Cowan et al., 1999; Bebko and Ricciuti, 2000). Children with ASD generally display lower rates of rehearsal when compared to TD peers, and they tend to rely on rote memory abilities (Hermelin and O’Connor, 1975; Farrant et al., 1999; Bebko and Ricciuti, 2000).

Much of the previous research on memory and strategy use has relied on chronological age as a relevant comparison variable among different samples of children, such as TD children and children with intellectual disabilities. Bebko and Luhaorg (1998) suggest that the poor use of rehearsal strategies by children with ASD compared to same-aged peers may be explained, at least in part, by the fact that rehearsal strategies are primarily a verbal process. Since a delay or lack of language acquisition is a common feature in ASD (indeed, it is often the first recognized symptom; Lord and Luyster, 2009), it is not surprising that the acquisition of language-based memory strategies would correspondingly be delayed. Bebko et al. (2014) proposed a model in which they argued more generally that chronological age should be regarded as more of a summary descriptor variable than a predictor. That is, age is simply a stand-in that marks the typical development of underlying skills, such as language proficiency and metamemory, and it is these skills that are more specifically related to the development of rehearsal strategies and the subsequent benefits to recall performance. This de-emphasis on age and a further focus on the underlying precursors of strategy use is particularly pertinent for special populations, for whom chronological age is often delinked from the development of these underlying predictors (Bebko et al., 2014).

Research with children who are deaf provides support for the use of more direct predictors of strategy use than chronological age. Similar to children with ASD, children who are deaf demonstrate a lag in the age of emergence of rehearsal strategies. For these children years of meaningful language experience (Bebko and McKinnon, 1990), language proficiency, and the automatization of language skills (Bebko et al., 1998) have been found to be significant predictors of rehearsal use and chronological age was not. These findings suggest that comparisons between typically and atypically developing children based on global concepts like age can misrepresent the developing memory capabilities of special populations: underlying variables such as language proficiency are more meaningful predictors.

Similar to rehearsal, metamemory is a language-loaded process. Metamemory refers to the awareness of one’s own memory, including the processes by which individuals are able to reflect on their memory skills, and the manner by which they use this knowledge to regulate their learning (e.g., Flavell, 1979; Bebko et al., 2014). Bebko and Luhaorg (1998) suggest that children’s growing awareness of their own memory skills and limitations allows them to understand more clearly the role of strategies in assisting recall on difficult tasks. Cavanaugh and Borkowski (1979) found that metamemory can play an important role in the successful training of rehearsal strategies, and it can predict the maintenance of trained rehearsal strategies. In general, metamemory knowledge has been linked to the spontaneous use of rehearsal strategies in TD children (e.g., Weed et al., 1990; Short et al., 1993; Bebko et al., 2014), although findings have been somewhat equivocal across studies.

Focusing on rehearsal strategy use in children with ASD, Joseph et al. (2005) found that while there were no impairments in recall when participants were given a sequence to rehearse, they displayed poorer performance on tasks that required them to generate sequences to rehearse themselves. As a result, Joseph et al. (2005) suggest that observed impairments in rehearsal strategy use in this population may be due to a more general impairment in self-monitoring, or metacognitive awareness. Interestingly, Wojcik et al. (2014) found that adolescents with a diagnosis of ASD displayed no impairments in metacognitive ability as measured by their ability to gage their own memory performance, and manage their time when learning new word pairs. However, when performance on metamemory tasks was analyzed in a study by Wojcik et al. (2013), impairments were found in trials that tapped into certain subdomains, such as metamemory associated with episodic memory, and not semantic memory.

In their study of metamemory in ASD, Farrant et al. (1999) outlined five classes of metamemory knowledge: (1) knowledge that mental states exist, termed “existence”; (2), the knowledge of distinct processes, i.e., guessing, knowing, dreaming; (3) “integration,” or knowledge that such distinct processes are related and interactive and together create “the mind”; (4) the knowledge that a number of variables help to influence acts of cognition, such as the abilities of a person or the size of memory span; and (5) “cognitive monitoring,” or the ability to measure the status of one’s own cognitive system and to utilize such information to direct behavior on cognitive tasks. Several researchers have hypothesized that it is cognitive monitoring that is associated with the difficulties children with ASD have in memory tasks, namely in the initiation and use of strategies to aid cognitive performances. Interestingly, Farrant et al. (1999) matched their participant groups on language ability, thus offsetting the delay in language acquisition often observed in children with ASD. Their findings that the children with ASD were not impaired relative to a comparison group in any of the metamemory tasks administered are consistent with the present focus on the role of underlying skills like language level in the emergence of rehearsal. Although metamemory abilities were not found to be impaired, Farrant et al. (1999) nonetheless found that children with ASD demonstrated difficulty in the initiation and use of memory strategies, and did not employ these skills at the same level as their peers.

Extending the link between metamemory and language skills further, metacognition, of which metamemory is a part, has been found to be positively correlated with verbal intelligence in TD children (Schneider et al., 2004), and children’s language abilities have been found to be predictive of metamemory function over time (Lockl and Schneider, 2007). Lockl and Schneider (2006) suggest that language acquisition is an important precursor to the development of metacognition in TD children. In particular, they suggest that the acquisition of “mental verbs,” or vocabulary that allows children to reason about the mental state of individuals that are not present, is predictive of metamemory abilities later on in development. Similarly, Cherney (2003) suggests that children’s metacognitive skills and memory abilities increase as language develops and the child becomes better able to understand words used to describe mental states.

The primary goal of the present study was to determine whether metamemory knowledge is predictive of verbal rehearsal and memory performance in individuals with ASD. Additionally, given the links between metamemory and language, we sought to examine the role that language proficiency plays for those with ASDs. In an earlier study examining the relationship among language proficiency, metamemory function and strategy use in TD children, we found that language proficiency was a strong predictor of strategy use, and also accounted for the relationship between metamemory and strategy use (Bebko et al., 2014). We expect that children with ASDs will display a similar pattern of results when language proficiency and metamemory knowledge are used as predictors of rehearsal and memory performance.

Therefore, consistent with the previous literature on limited strategy use among populations with ASDs, we expected to find fewer spontaneous cumulative rehearsers among children with autism relative to a non-autism comparison group. However, regardless of group, rehearsers were expected to recall more than non-rehearsers. In term of metamemory, we hypothesized that children who spontaneously use a verbal rehearsal strategy would have significantly higher metamemory scores than those who do not spontaneously rehearse. We expected this relationship to hold for both participant groups.

Given that metamemory is a highly language-loaded process, we also hypothesized that scores on a metamemory questionnaire would be significantly correlated with language proficiency scores for both groups. As a result, we expected that both metamemory and language skills would be a strong predictors of rehearsal use and, as a result, of recall performance. This finding would generalize the findings and model in Bebko et al. (2014) by extending them to children with developmental difficulties.

## MATERIALS AND METHODS

### PARTICIPANTS

The participants in this study included two groups. Membership in the ASD group was based on the nature of the child’s school program (i.e., education classes for children with ASD), and on the results of diagnostic and standardized testing conducted previously in participants’ schools or clinics by registered health care professionals (i.e., psychologist, pediatrician). The ASD group included 21 children with a previous diagnosis of ASD according to DSM-IV diagnostic criteria or earlier versions (American Psychiatric Association [APA], 1994), and Verbal IQ ≥ 40, and verbal mental age (VMA) >4 years. The second group consisted of 21 TD children, who were assumed to have average intellectual functioning, or children with an intellectual disability (VIQ 40–70, VMA >4 years) with no known organic etiology. This blended group was used as a best match to the group with ASD, as the full range of ASDs encompasses children with and without associated intellectual disabilities. Groups were individually matched on chronological and VMA, and, as seen in **Table 1**, the two samples were well-matched on most variables with the exception of gender. However, previous literature has shown no significant differences in strategy emergence by gender (e.g., Flavell et al., 1966; Bebko, 1979).

**Table 1:** Participants – sample composition.

	ASD sample	(*n =* 21)	Comparison sample (*n =* 21)
**Gender**
Males	21	8
Mean chronological age (range)	10.57 years (7–16.58)	10.67 years (5.80–14)
Mean verbal mental age (range)	7.44 years	7.64 years
	(4.20–11.25)	(3.94–10.33)
# Children with VIQ <70	10	11
# Children with VIQ >70	11	10
Bankson language scores mean (SD)	95.12 (21.43)	91.33 (26.15)

Intelligence scores for the ASD group and those in the comparison group with intellectual disability were obtained from school or clinic reports based on assessments done within the past 2 years. In each case, scores were derived from the edition of the Wechsler (WISC) or Stanford–Binet Intelligence tests current at the time of their assessment. Verbal IQ (VIQ) scores, as opposed to Full Scale IQ (FSIQ) or Performance IQ (PIQ) scores were used to match the two participant groups, as typically there are large discrepancies between individuals with ASD’s performance on VIQ and PIQ subscale, with the PIQ typically superior to the VIQ (e.g., Wing, 1989). With such large differences on these subscales, any calculated FSIQ is unreliable (Sattler, 2001). In some earlier studies, children with ASDs were often matched to comparison groups on the basis of PIQ, typically their strongest skill (e.g., Boucher and Warrington, 1976; Ameli et al., 1988). The participants were then often tested on tasks that involved verbal components, thereby biasing against the group with ASD. A more accurate comparison would be to match participants on the basis of VIQ. Then, if differences are still found, it is more certain they are due to characteristics of ASD. In addition, language abilities are thought to be linked to one of the dependent variables in the present study (rehearsal use); therefore controlling VIQ and VMA is more important than performance mental age (PMA).

### APPARATUS AND PROCEDURE

The current study involved two testing sessions. Session 1, which was adapted from earlier studies (viz., Bebko, 1984; Bebko and Ricciuti, 2000), included familiarizing participants to the procedure, a serial recall task, and a brief language assessment. Session 2 took place 1–3 days later.

#### Session 1 – serial recall trials

***Materials.*** Stimuli for the serial recall task involved 12 different colored photographs (6 cm × 8 cm) of common objects (ball, chair, chips, coat, crayons, cup, hands, knee, scissors, shoes, spoon) that were mounted on white bristol board cards (6 cm × 10 cm). Two sets of stimulus cards were used, one set as stimuli, and the other set as response cards.

***Design and procedure.*** Each child participated in six data trials, consisting of two blocks of 3, 4, and 5-picture arrays, presented in ascending order within each block. Varying lengths of arrays were included to avoid frustration and ceiling effects. Each of the 12 stimuli occurred no more than once within a given sequence and an approximately equal number of times across arrays. An adjacent pair of pictures could not recur in consecutive arrays. The stimulus cards were exposed one at time for 3 s each. When all were exposed, they were then turned upside-down and an unfilled 15-s delay began before recall. Throughout stimulus presentation and the delay, the response cards were covered. After the delay, the response cards were uncovered. Participants were then asked to choose the response cards they had been shown, and arrange them in the same order they had been presented.

Initially, practice trials of two stimuli each were used to familiarize the child with the task, and to ensure that they understood the instructions. In order to proceed, the child was required to complete a minimum of two trials of two stimuli recalled in correct order. Verbal reinforcement for effort was used intermittently throughout, regardless of correct or incorrect responses.

Following the recall trials, the child was reinforced for doing well on the task, and then was asked how s/he remembered the information. Self-reports of “memory tricks” (strategies) were recorded. Unclear responses were followed with up to two neutral clarification probes (e.g., “tell me more about that”; or “I don’t exactly understand; what if I showed you apple, hands, spoon, how would you remember them?”).

During display, delay, and recall phases of the serial recall trials, the child’s behavior was carefully observed and videotaped. Serial recall scores, free recall scores and behavior observations/verbal reports were all coded. Serial recall scores represented the number of correct items recalled in the correct serial position. The sum of all the scores on individual trials was calculated, with a maximum score of 24. Free recall scores were the overall number of items correctly recalled, disregarding serial position errors (maximum score of 24). Behavior observations and the verbal reports were coded using well-established criteria based on previous studies (e.g., Bebko, 1984; Bebko and Ricciuti, 2000; Bebko et al., 2014) and derived from Flavell et al. (1966). Overt signs of cumulative rehearsal included verbal cumulative rehearsal (e.g., “apple, ball, shoes…apple, ball, shoes”), recognizable lip movements, rhythmic body movements (e.g., finger pointing, rhythmic head movements, eye movements). Clear self-reports of rehearsal when the children were asked about “memory tricks” were also accepted as an indication of rehearsal, on the assumption that the children would not likely report such a strategy if they had not been using it. In order for a participant to be identified as a rehearser, the above behaviors must have been observed on ≥2 trials, or the participant must have clearly reported using rehearsal (or both). Inter-rater reliability was calculated on the classification of 100% of the participants, and 98% agreement was obtained between two independent raters. Any discrepancy was resolved by discussion. All classifications were made without knowledge of results of the language measure.

Following the completion of the serial recall task, the Bankson Language Scale (Bankson, 1977, 1990) was administered to all participants in order to obtain a language proficiency score. For the current study, only the expressive scales (semantic knowledge, morphological, and syntactic rules) were used, as the last two categories of the scale (auditory memory, auditory sequencing/discrimination) rely directly on short term memory skills, and the purpose was to get a measure of language independent of short term memory. The Bankson was given individually to all participants. Concurrent validity has been established with other standardized language measures, such as the Peabody Picture Vocabulary Test, *r* = 0.54, Boehm Concept Test, *r* = 0.62, Test of Auditory Comprehension on Language, *r* = 0.64 (Bankson, 1977) and Test of Language Development-Primary, *r* = 0.64 to 0.97 (Newcomer and Hammill, 1997).

#### Session 2 – metamemory task

***Materials.*** Metamemory was assessed using a series of seven questions adapted from previous studies examining metamemory in children (Flavell and Wellman, 1977; Brown, 1978; Bebko et al., 2014). Previous literature highlights that when assessing one’s metacognition, it is important to examine an individual’s metacognitive abilities related to the person, the task, and memory strategies utilized. In particular, research demonstrates that metamemory judgments require: (1) knowledge of the self, or one’s ability to estimate one’s own memory capabilities (e.g., memory span; Flavell et al., 1970; Brown, 1978); (2) awareness of task difficulty (categorization versus non-categorization; Kreutzer et al., 1975; Salatas and Flavell, 1976); and (3) the degree to which an individual is aware of when they need to use strategy (Flavell and Wellman, 1977). Thus, in the present study the metamemory questions examined participants’ knowledge of the usefulness of categorization, the effect of number of stimuli to remember, knowledge of his/her own memory span, and his/her knowledge of memory strategies. Photograph stimulus cards used in the serial recall task were used as aids. In terms of validity, the majority of studies have shown evidence of moderate to strong internal validity of individually administered metamemory questionnaires (e.g., Flavell et al., 1970; Kreutzer et al., 1975). However, Belmont and Borkowski (1988) noted that the validity of metamemory questionnaires is developmentally sensitive, that is, the internal consistently varies, as samples of children gets older. Although the present questionnaire was not intended to be a unitary measure, as it evaluates several different components of metamemory, the overall measure nonetheless yielded a Cronbachs’ α = 0.66, which indicates minimally acceptable internal consistency (e.g., DeVellis, 2003).

***Design and procedure.*** Session 2 took place 1–3 days after Session 1. Similar to Session 1, Session 2 was videotaped so that incomplete or unclear recording could be revisited. Each participant was evaluated on four categories of metamemory knowledge. The first was knowledge of task variables – semantic, which assessed participant’s knowledge of the usefulness of implicit categories (Questions 1, 2). In these two questions, children were presented with two groups of four picture cards; one group contained four related items in two categories (e.g., two clothing and two food items), while the other group showed four completely unrelated items. The child was then asked which group of four cards was easier to remember (Question 1) and was asked for justification of his/her response (Question 2). The second category of metamemory was knowledge of task variables – quantitative, in which participants were tested on his/her knowledge of the number of items they could remember (Questions 3, 4). This involved the child being presented with two groups of stimuli, one containing four cards, and the other containing eight cards. The child was then asked which group would be easier to remember, and why. Knowledge of the self (Questions 5, 6) examined the accuracy of knowledge of his/her own memory span. The child was shown a group of 4 and a group of 8 pictures, and then asked how many s/he could remember from each set. After the participant offered a prediction, actual performance on the task was then determined. Lastly, participant’s knowledge of strategies (Question 7), or the participant’s knowledge of the need for memory strategies was examined. The participant was asked what s/he would do if there was something difficult to remember.

The experimenter recorded all responses, and each metamemory question was scored on a two-point scale. Two points were credited if the response reflected the memory-reducing difference between the stimuli for Questions 1 (categories) and 3 (reduced number). For Questions 2 and 4, memory-reducing rationales received full credit, i.e., pointing out that the grouped stimuli are related, and that four stimuli are easier than eight because there were fewer. However, a clear rationale was considered to compensate even if the less obvious response was chosen (e.g., choosing the larger group, because several of them are favorite things and easy to remember.) For Questions 5 and 6, predictions within one item of actual recall for the four picture trial or within two items for the eight picture trial were given two points; predictions within two items for the four-picture trial, or within three, four or five items of actual recall for the eight- picture trial, were awarded one point. On the last metamemory question, clear knowledge of internal or external strategies was awarded the two points, whereas vague responses were given one point, and a response of “I don’t know,” or no response, was scored as 0. Participants’ total scores across questions were divided by the total metamemory score of 14 to obtain proportion scores which were used in the main analyses. Metamemory data for one of the participants with ASD was lost due to experimental error.

## RESULTS

A oneway analysis of variance (ANOVA) on scores from the Bankson measure examining language proficiency indicated no groups differences between the ASD (mean = 95.12; SD = 21.43) and non-ASD samples (mean = 91.33; SD = 26.15), *F*(1,36) = 0.23, *p* = 0.634, which further corroborates the matching of the samples, as indicated in the similar VMA scores. A strong correlation between the VMA and Bankson language proficiency scores, *r*(38) = 0.78, *p* = 0.0001, indicates considerable shared variance, as would be expected.

**FIGURE 1:**
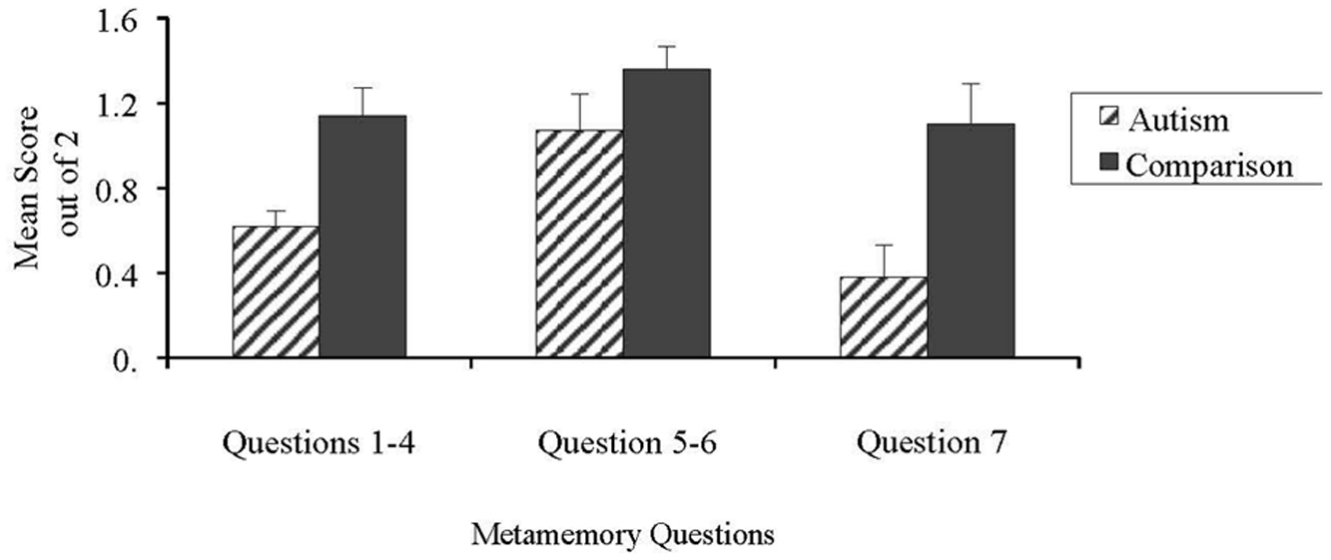
**Children with ASD versus comparison group’s performance on the three subsets of metamemory questions (knowledge of task, knowledge of self, knowledge of strategies)**.

**FIGURE 2:**
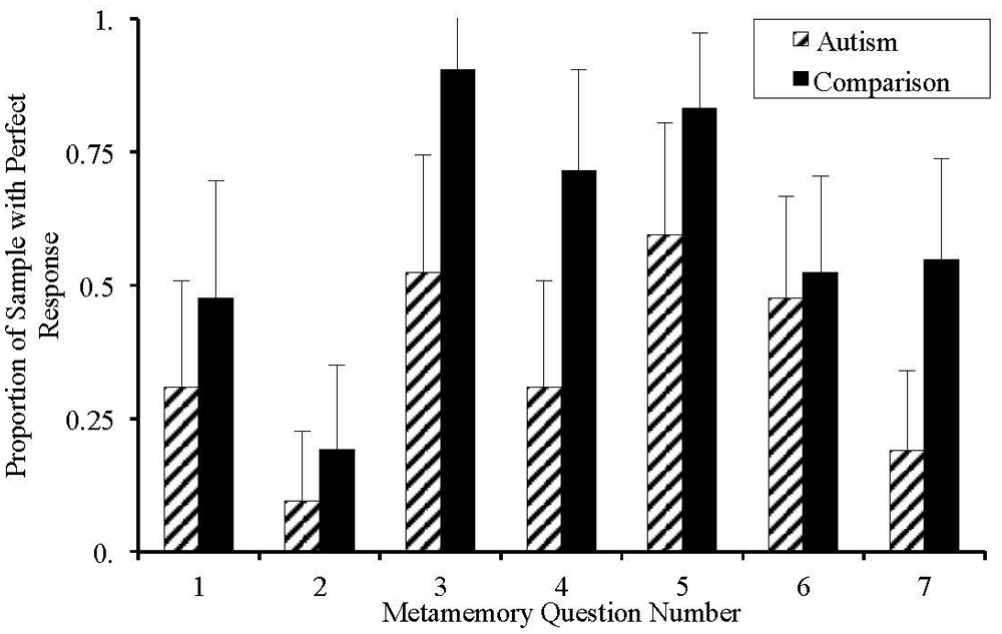
**Children with ASD versus the comparison group’s performance on each individual question of metamemory test**.

Next, the results from the metamemory questionnaire are presented first, followed by the results from the serial recall task. Performance by both groups on the subsets of metamemory questions is summarized in **Figure 1**, and by individual question in **Figure 2**.

### KNOWLEDGE OF VARIABLES: SEMANTIC AND QUANTITATIVE

Questions 1 through 4 on the metamemory questionnaire focused on the nature of the materials being presented. Specifically, participants were tested on their knowledge related to the similarity of stimuli (e.g., stimuli presented in categories versus random stimuli), and the quantity of stimuli (e.g., fewer pictures versus many pictures). A one-way ANOVA was conducted to compare scores between groups for the first four metamemory questions combined. As shown in **Figure 1**, there was a significant group effect, with the comparison group performing better than the group with ASD, *F*(1,40) = 11.64, *p* < 0.01.

### KNOWLEDGE OF SELF

Questions 5 and 6 of the metamemory questionnaire dealt specifically with knowledge of one’s own memory capacity by predicting how many pictures would be remembered out of groups of 4 and 8, followed by testing for actual recall. Overall, a one-way ANOVA showed that groups did not differ significantly in their estimations relative to their performance, *F*(1,40) = 1.99, *p* = 0.166.

### KNOWLEDGE OF STRATEGIES

The final question of the metamemory questionnaire asked participants what they would do to help themselves remember difficult information. Results from a one-way ANOVA showed that the comparison group demonstrated significantly greater knowledge of strategies than the children with ASD, *F*(1,40) = 8.65, *p* < 0.01.

### OVERALL METAMEMORY SCORE

The overall metamemory questionnaire yielded a total score out of 14. The mean percentage total score for children in the comparison group was 59.7% and for the children with ASD was 35.8%, a difference that was highly significant, *F*(1,40) = 12.28, *p* < 0.01.

#### Serial recall task

**FIGURE 3:**
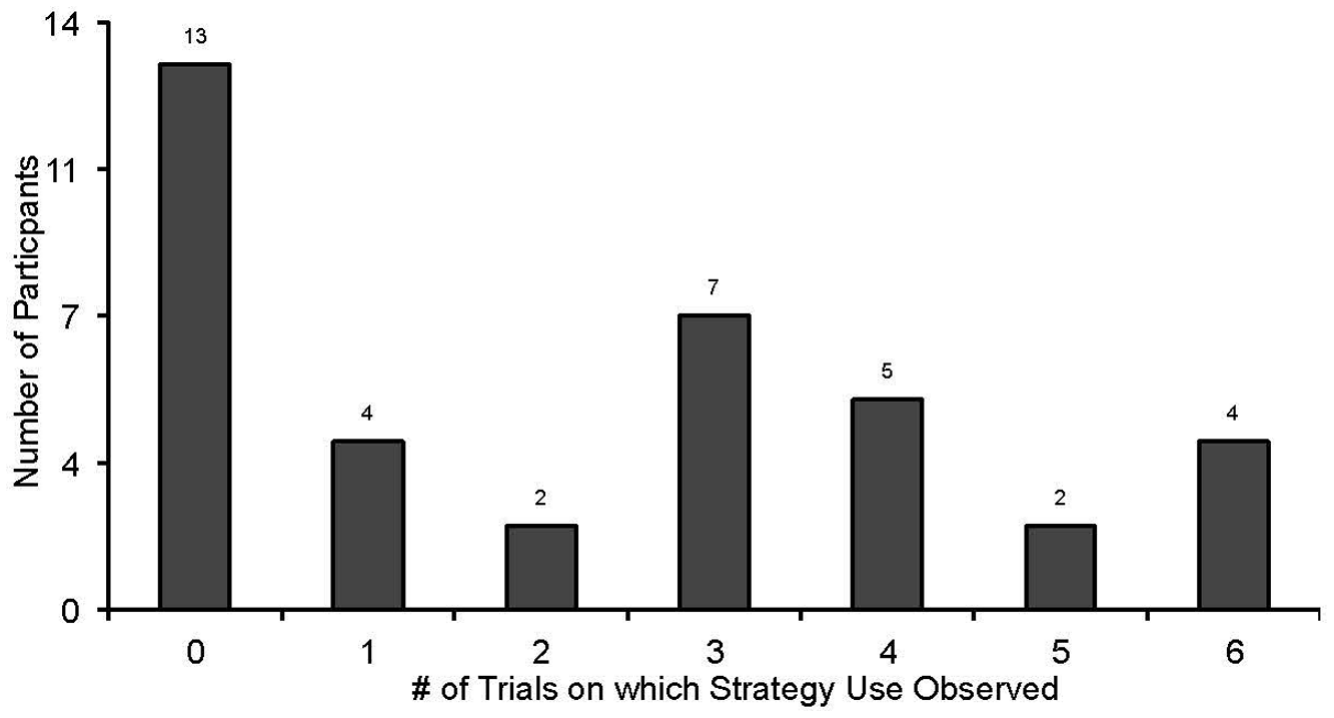
**Number of participants using a rehearsal strategy during the serial recall task by number of trials on which rehearsal was observed**.

***Rehearser classification.*** A wide variety of behaviors associated with strategy use were observed during stimulus presentation and the 15-s delay period prior to recall (see Materials and Methods). The number of trials on which rehearsal was observed is shown in **Figure 3**. The rehearser classification cutoff of two trials corresponds to one of the lowest points in the distribution across trials, thus decreasing the likelihood of misclassification errors based on behavioral observation alone. There are two potential reasons why the participant would not be observed using strategic behavior: (1) the child simply was not rehearsing; or (2) the child was rehearsing, but covertly, as has been shown to occur in previous studies (e.g., Flavell et al., 1966; Bebko, 1984). Only two participants were identified as rehearsers from their reports alone, based on the assumption that if they were able to report using the strategy then they were covert strategy users. The other eight children who reported using a strategy also demonstrated observable behavior on at least two trials.

For the group with ASD, 6 out of 21 participants (28.6%) were classified as rehearsers, compared to14 of the 21 participants in the comparison group (66.7%), despite being matched on VMA. This difference was significant, *z* = 2.47, *p* < 0.05. Additionally, participants classified as rehearsers (regardless of group), performed significantly better on the metamemory questionnaire than non-rehearsers*, F*(1,40) = 21.79, *p* < 0.001; however, the interaction was not significant.

***Recall performance.*** Recall performance was examined in two 2 (rehearser, non-rehearser) × 2 (autism, comparison group) × 6 (repeated measure: list length) ANOVAs for serial recall and free recall scores. Overall, rehearsers were found to have performed significantly better than non-rehearsers for both serial recall, *F*(1,32) = 34.95, *p* < 0.001, and free recall, *F*(1,33) = 5.01, *p* = 0.032 (**Table 2**). For serial recall, the rehearser group × list length interaction was also significant, *F*(5,160) = 2.55, *p* = 0.030, as the difference between the rehearser groups increased as list length increased; for free recall, there were no significant interaction effects. Of note, there was no significant main effect for group for either serial recall, *F*(1,32) = 0.031, *p* = 0.86, or free recall, *F*(1,33) = 0.261, *p* = 0.552 (**Table 2**). The interactions were also not significant (all *p* > 0.453).

**Table 2:** Rehearsers versus non-rehearsers serial recall and free recall performance.

	Serial recall *M* (SD)	Free recall *M* (SD)
**Autism group**
Rehearsers (*n =* 6)	0.81 (0.19)	0.88 (0.19)
Non-rehearsers (*n* = 15)	0.37 (0.21)	0.64 (0.25)
Total (*n* = 21)	0.49 (0.28)	0.70 (0.25)
**Non – autism group**
Rehearsers (*n* = 14)	0.83 (0.15)	0.94 (0.06)
Non-rehearsers (*n* = 7)	0.41 (0.23)	0.76 (0.18)
Total (*n =* 21)	0.69 (0.27)	0.88 (0.14)

***Predictive variables.*** A logistic regression analysis was used to examine the strength of metamemory scores and language skills as predictors of which participants would be spontaneous strategy users. First, scores of all the participants on the metamemory questionnaire were entered into the model independently. The logistic regression results showed that the scores on the metamemory questionnaire were highly significant predictors of spontaneous rehearsers (**Table 3**). Metamemory scores successfully classified 74% of the participants as strategy users or non-users. Logistic regression analyses were also completed separately for each diagnostic group. Scores on the metamemory questionnaire successfully classified 76% of the comparison group as rehearsers or non-rehearsers, which proved to be nearly a significant predictor (*p* = 0.066). Metamemory scores were significant predictors of rehearsers in the ASD group (*p* = 0.044), correctly classifying 76% of the cases with ASD. As expected, scores on the metamemory questionnaire were also highly correlated with serial recall performance across both groups (autism group, *r* = 0.489, *p* < 0.05; comparison group*, r* = 0.576, *p* < 0.01) and across all participants (*r* = 0.583, *p* < 0.01).

Since the metamemory questionnaire is a highly language-based measure, a Pearson correlation was calculated between the Bankson and the metamemory questionnaire data, and a significant correlation was found, *r* = 0.366, *p* < 0.05. However, with only 13% of the variance accounted for, it appears that metamemory and language, although related, remain quite separate contributors to the emergence of strategy use.

**Table 3:** Summary of the regression analysis examining metamemory as a predictive variable of spontaneous rehearsal use.

	*B*	*SE*	*Wald*	*% correctly classified*
**Group**
ASD	6.19	3.08	4.04*	76.2
Comparison	8.2	4.46	3.4*	76.2
Total	7.55	2.45	9.5*	73.8

*p < 0.01.

We examined the metamemory and language relationship further, using a hierarchical logistic regression model, with metamemory entered first, followed by scores on the Bankson Language Scale. Overall, both metamemory and Bankson scores remained significant predictors of spontaneous strategy use, Wald’s statistic = 5.02, *p* < 0.05 for metamemory, and 7.40, *p* < 0.01, for the language variable. Together, they correctly predicted 89.5% of the participants as rehearsers or non-rehearsers (see **Table 4**). This evidence provides corroborative support that both metamemory and language proficiency, while separate contributors, are both important predictors for the emergence of spontaneous rehearsal.

**Table 4:** Summary of hierarchical regression examining metamemory and language proficiency as predictive variables of spontaneous rehearsal use.

	*B*	*SE*	*Wald*	*% correctly classified*
**Variable**
Metamemory	6.36	2.84	5.02*	
Language	0.09	0.03	7.39*	
Constant	12.0	4.01	9.06*	
Total model				89.5

*p < 0.01.

Finally, we considered the relevance of two potential covariates among these samples, gender and age, as the groups differed widely in their gender composition (ASDs are approximately four times more common in males than females), and there were wide age ranges in both groups. (VMA was not evaluated due to its very high shared variance with language scores (61%), reported above.) As noted in Section “Introduction,” chronological age is a global concept representing physical development that can mask the development of the more proximate predictors of cognitive skills, particularly in clinical populations, so was not of interest. However, the matching process here resulted in ranges of age spanning nearly 10 years in both samples, which represents a considerable developmental period. The logistic regression was repeated entering these age and gender variables as covariates in Block 1. The resulting model was not significant χ^2^(2) = 0.734, *p* = 0.734, and neither age, Wald = 0.493, *p* = 0.482, nor gender, Wald = 0.259, *p* = 0.611 were significant. When Bankson and metamemory scores were then entered, the logistic regression model became highly significant, χ^2^(4) = 27.41, *p* < 0.0001. Gender and age remained non-significant covariates, both *p* > 0.679, while the language variable and metamemory remained as strong predictors. The significance level for language was Wald = 6.61, *p* = 0.01, and for metamemory was Wald = 3.51, *p* = 0.06, the latter indicating marginal significance; the accuracy of prediction of rehearsers or non-rehearsers by the model remained unchanged at 89.5% with or without the covariates.

## DISCUSSION

This study was an examination of spontaneous verbal rehearsal strategies and variables contributing to their use by children with ASD, a group previously observed to be relatively impoverished in their use of self-generated strategies when processing information. Previous work from our lab (Bebko et al., 2014) found support for a model in which metamemory and language skills were identified as strong predictors of rehearsal strategy use in TD children. In the present study we examined whether the same variables could predict the emergence of verbal rehearsal in children with ASD. We found that the model generalized well, in that comparable relationships were found between rehearsal use and its predictor variables for children with ASD and the comparison group.

Consistent with previous studies (e.g., Bebko and Ricciuti, 2000; Joseph et al., 2005), the rate of spontaneous rehearsal use was much lower in children with ASD, with fewer than half the number of rehearsers among the children with ASD versus the comparison group (approximately 1/3 versus 2/3 of the samples, respectively), despite the groups being matched on VMA and chronological age. Moreover, for both groups, those children who did rehearse showed comparable levels of recall, and at much higher levels than non-rehearsers. While rehearsal is most commonly associated with tasks requiring ordered recall, the benefit of rehearsal was seen for both serial and free recall.

In terms of predicting rehearsal, the findings of a strong relationship between metamemory performance and strategy use in the two groups tested here is important for two reasons. On the one hand, the association between metamemory and rehearsal use has been somewhat equivocal historically in the memory literature. However, the present results corroborate those in Bebko et al. (2014) that showed a strong predictive relationship. At the same time, the replication and extension of these findings in a clinical population who are often weak in strategy use, lends additional credence to this predictive relationship.

Going beyond the metamemory-rehearsal relation, the strong predictive role of language proficiency in rehearsal use was also supported. Given that metamemory is typically examined using verbal protocols, the relationship is a logical one. However, the finding of a comparable relationship in a clinical sample where language deficits are common, and a diagnostic feature of the disorder, speaks to the strength of the role of language proficiency in the language – metamemory – rehearsal use – recall constellation. Knowledge of only the metamemory scores and language scores of participants was sufficient to accurately predict those who would be a rehearsal user for 90% of the combined diagnostic groups.

The findings of poorer metamemory skills in children with ASD compared to matched peers, is consistent with the limited metamemory and ASD literature to date (Farrant et al., 1999; Wilkinson et al., 2010; Wojcik et al., 2014). Furthermore, the work by Wilkinson et al. (2010) suggests that any differences in memory performance that we found when comparing children with ASD to their peers are likely to lessen as more skills are gained. Specifically, they compared metamemory skills of children and adults with ASD, and found an apparent developmental effect, where memory awareness was relatively poor for children, but only subtly different for adults with ASD compared to peers.

The metamemory differences in the ASD group seemed mostly associated with their knowledge of how task variables can impact their memory performance (Questions 1–4), and the awareness of the need to use a memory strategy in challenging memory situations (Question 7). Success on most memory tasks requires the quick recognition of the need to use a strategy and flexibility in the choice of strategies. A number of studies have identified reduced processing speed in many children with ASD (e.g., Oliveras-Rentas et al., 2012; Hedvall et al., 2013) and others have found less flexible memory strategy use during recall tasks. Consistent with their lower performance on the knowledge of categories questions (Questions 1 and 2) in our metamemory interview, Minshew et al. (1992) and Minshew and Goldstein (1993) found that children with ASD had difficulty grouping material into categories. It is not a memory capacity issue, as a number of studies (e.g., Russell et al., 1996) have found intact working memory capacity in ASD, but difficulty on tasks that involve both working memory and simultaneous strategies for the storage of that information. Therefore, children with ASD are likely to be more affected by the specific task used to test memory skills. In Bebko and Ricciuti (2000), significantly more spontaneous rehearsal use in children with ASD was observed when task constraints were lessened. When the participants were given control over how long to study the stimuli in a recall readiness task, more spontaneous rehearsal use was observed compared to an experimenter-controlled condition. Considering the group with ASD’s relatively intact knowledge of their own abilities, as demonstrated by less impaired performance on the corresponding metamemory questions in the present study (Questions 5 and 6), recall readiness conditions not only allow for increased time for processing, but the paradigm may also play into the relative strengths of the group with ASD in estimating their own knowledge.

The finding of overall delayed metamemory skills may provide a partial explanation for the theory of mind (ToM) deficit in ASD identified by Baron-Cohen and others (e.g., Baron-Cohen et al., 1985; Baron-Cohen, 1995). The inability to infer the mental states of other people (e.g., their beliefs, desires, intentions) that is a hallmark of a ToM deficit may begin from, or be associated with, an impairment in the awareness of one’s *own* cognitions (i.e., metacognition). This relationship was also suggested by Frith (1989). Lockl and Schneider (2007) investigated the development of both ToM and metamemory in TD children and found a moderately strong developmental relation, although they came to an alternate conclusion, that early ToM competencies may be a precursor of subsequent metamemory development.

Our findings of the association between language and metamemory skills, here with ASD, and by Bebko et al. (2014) with TD children, parallel similar findings in Lockl and Schneider (2007) that language skills are critical precursors of metamemory skills. The present study expands those links to rehearsal strategy use and, in doing so, generalizes the Bebko et al. (2014) findings of the linkages among language development, metamemory development, and the emergence of verbal rehearsal in TD children to a population of children with known language impairments. However, these relationships are in need of further research in children with ASD. In addition, although the gender split between our groups proved to be a non-significant co-variate in the present analyses, better balanced samples would provide a more comprehensive test of that issue. Similarly, variables controlled or otherwise not examined directly in the current study, such as aspects of intelligence, may contribute to the development of strategies including rehearsal.

Memory and metamemory research in ASD have important pedagogical implications: both in specific learning contexts and in everyday problem-solving scenarios. If a child is struggling in the classroom, the focus should perhaps move away from the content of the material, to providing an appropriate strategy for learning. It would be important to highlight metamemory components as well, such as when to use a specific strategy, showing the results explicitly of using versus not using the strategy, and discussing other contexts in which the strategy might be used. This type of additional support may enhance the learning of various strategies, as well as enhance the generalizing of them to new learning situations, which is a significant challenge for children with ASD.

## Conflict of Interest Statement

The authors declare that the research was conducted in the absence of any commercial or financial relationships that could be construed as a potential conflict of interest.
